# Comparison of novel and established caries diagnostic methods: a clinical study on occlusal surfaces

**DOI:** 10.1186/s12903-021-01465-8

**Published:** 2021-03-05

**Authors:** Friederike Litzenburger, Gerrit Schäfer, Reinhard Hickel, Jan Kühnisch, Katrin Heck

**Affiliations:** Department of Conservative Dentistry and Periodontology, University Hospital, Ludwig-Maximilians-University of Munich, Munich, Germany

**Keywords:** Dental caries, Occlusal caries, Occlusal caries detection, Diagnostic imaging, Near‐infrared transillumination, Bitewing radiography, Laser‐fluorescence, Alternating current impedance spectroscopy, Sensitivity, Specificity

## Abstract

**Background:**

The purpose of this prospective clinical diagnostic study with validation was to compare the diagnostic accuracy of near-infrared transillumination (NIRT), laser fluorescence measurement (LF), alternating current impedance spectroscopy (ACIS) and their combinations as adjunct methods to visual examination (VE) for occlusal caries detection using a hybrid reference standard.

**Methods:**

Ninety-six first and second non-cavitated permanent molars from 76 individuals (mean age 24.2) were investigated using (VE) (ICDAS) and bitewing radiography (BWR), as well as NIRT, LF and ACIS. The findings of BWR and NIRT were evaluated by two examiners while the other examinations were conducted by one calibrated dentist. The hybrid reference standard consisted of non-operative validation based on the results of VE and BWR and operative validation. Statistical analysis included cross-tabulations, calculation of sensitivity, specificity and area under the receiver operating characteristic curve at three diagnostic thresholds: caries in general, enamel caries and dentin caries.

**Results:**

NIRT, LF and ACIS exhibited high sensitivity for caries in general [1.00 (1.00–1.00), 0.77 (0.65–0.88), 0.75 (0.63–0.87)) and for dentin caries (0.97 (0.91–1.03), 0.76 (0.76–0.90), 0.64 (0.47–0.80)]. Sensitivity values for enamel caries were weak (0.21, 0.11, 0.37). Specificity values did not fall below 0.65 (NIRT) for all categories and methods, except for NIRT at the caries detection threshold (0.27). A combination of LF and ACIS with VE improved the diagnostic performance at the overall and the enamel caries threshold. The other methods showed fair to excellent discrimination at the overall caries threshold (NIRT 0.64, LF 0.89 and ACIS 0.86) and acceptable discrimination at the dentin caries threshold (NIRT 0.82, LF 0.81 and ACIS 0.79). AUROC for enamel caries exhibited the weakest discrimination. Accuracy was 65.6% for VE, 69.8% for BWR, 50.0% for NIRT, 53.1% for LF and 74.0% for ACIS. Reliability assessment for BWR and NIRT showed at least substantial agreements for all analyses.

**Conclusions:**

The methods, NIRT, LF and ACIS, revealed different potential but no impeccable performance for occlusal caries detection. All are suitable instruments to detect hidden carious lesion in dentin. As auxiliaries to VE, LF and ACIS showed an increase in diagnostic performance.

## Background

Dental caries is still a prevalent disease [1]. However, in industrialized countries, the prevalence of caries has declined, and lesion appearance has shifted towards a larger portion of non-cavitated caries [2–6]. Optimal caries management requires structured caries detection, assessment, and diagnostic procedures. Visual examination (VE) is recognized as the first method of choice due to its simplicity, acceptable validity and reliability, especially for early occlusal caries detection and assessment [3, 7, 8]. Besides, bitewing radiography (BWR) is frequently considered adjunct diagnostic method of choice because of its widespread availability in dental practices, complete imaging of the posterior region on one side of the jaw, visualization of caries extension in relation to the pulp and acceptable validity and reliability [9]. Aiming at limiting the exposure to ionizing radiation for dental diagnostic purposes, many X-ray-free diagnostic methods have been introduced on the dental market. Here, laser fluorescence measurement (LF), e.g. the DIAGNOdent (KaVo, Biberach, Germany), was first proven to be a valuable method for the evaluation of occlusal sites [10]. Since then, several other—mostly light-optical—devices have been developed and have received increasing clinical and scientific attention. Near-infrared light transillumination (NIRT) and alternating current impedance measurement (ACIS) have been recently introduced into dental practice [11–13], and only a few diagnostic studies have analysed their diagnostic performance for occlusal caries detection thus far [14–20]. A few clinical studies directly compared the diagnostic performances of these adjunct methods, highlighting their individual potential for occlusal caries detection at the enamel and dentin threshold [15, 21, 22].

The objective of this clinical study using a hybrid reference standard was to compare the diagnostic accuracy of NIRT, LF and ACIS alone and as adjunct methods to VE for occlusal caries detection in relation to the thresholds for overall caries, enamel caries and dentin caries. The null hypothesis was that all diagnostic procedures would exhibit similar diagnostic performance.

## Methods

This prospectively designed clinical diagnostic study was approved by the Ethics Committee of the Medical Faculty of the Ludwig-Maximilians University of Munich (Project Number 013-12).

### Sample size calculation

The assumed caries prevalence of the study population is approximately 50%. Aiming for a power of 80% and setting alpha to 0.05 with the null hypothesis for sensitivity (SE) and specificity (SP) at 0.5, which was supposed to increase to 0.7, 98 samples were calculated to be required [23].

### Eligibility of patients and teeth

The participants of this study were patients who came to the Department of Conservative Dentistry in Munich with the request for a dental examination and/or treatment from December 2012 to July 2014. Only healthy patients in generally good condition (ASA 1), with a minimum age of 12 years and a fully erupted permanent dentition were included. Further inclusion criteria were the presence of at least one molar without restoration, fissure sealing, orthodontic treatment, development defects or macroscopic cavitations. If these inclusion criteria were met, the patient was explained the study design and asked to participate (JK and FL). In case of a positive response, written informed consent was obtained. A further prerequisite for participation was the availability of bitewing radiographs which were not older than four months. New radiographs were only prescribed when there was a justifiable indication. These radiographs were analysed by the recruiting dentists (JK and FL) as part of the initial examination. The patients were informed about their findings, and all adequate therapeutic options were enumerated and offered.

### Clinical examination

Visual examination was performed using magnifying lights (magnification: 2.5×, focal distance: 300–550 mm, field of view: 67–115 mm) after a professional tooth cleaning after ~ 5 s of air-drying by two calibrated dentists (JK and FL). All results of the VE were discussed and evaluated among the examiners (JK and FL) during the same appointment. The findings were categorized according to the International Caries Detection and Assessment System II (ICDAS) for occlusal surfaces with the following relevant scores: sound, first visible change in enamel, distinct visible change in enamel and localized enamel breakdown without visible dentin or underlying shadow [7]. Photographs were captured to allow later reassessment of the surfaces.

### Digital bitewing radiography

Bitewing radiographs were acquired using an intraoral dental X-ray machine with a 203 mm tube (Heliodent DS, Sirona, Bensheim, Germany) including an X-ray field limitation (30 × 40 mm) with a CCD sensor (Intraoral II, sensor size 30.7 × 40.7 mm, Sirona, Bensheim, Germany) and an exposure time of 0.06 s at a cathode voltage of 60 kV and 7 mA of amperage. For the parallel technique, a sensor-holding system (XPP-DS Digital Sensor Holders for Sirona, Dentsply Rinn, Elgin, IL, USA) was used. Evaluation of all radiographs was conducted by two examiners (FL and KH) independently from each other and blindly from other diagnostic findings in a darkened room on a standard calibrated monitor according to the following criteria: no signs of various decalcification, translucence in enamel or translucence in dentin (Table 1). If the examiners made different diagnoses, then they re-assessed the corresponding radiographs, discussed their findings, and reached a consensus finding.

**Table 1:** Description of the criteria and threshold values for visual examination (VE) [51], bitewing radiography (BWR), near-infrared transillumination (NIRT), laser fluorescence measurement (LF) and alternating current impedance spectroscopy (ACIS) [25]

	VE		BWR		NIRT		LF		ACIS	
Sound	0	Sound surface	0	No radiolucency	0	No less translucent spots	0	0–12	0	0–20
Enamel caries	1	First visual demineralization in dried enamel	1	Radiolucency in the outer half of the enamel	1	First or established lesion restricted to the enamel, no visible translucent dentin	1	13–24	1	21–90
	2	First visual demineralization in dried enamel with a distinct change in moist enamel	2	Radiolucency restricted to the inner half of the enamel						
	3	Localized enamel breakdown without visible dentin								
Dentin caries	4	Underlying dark dentin shadow with or without enamel cavitation	3	Radiolucency restricted to the outer half of the dentin	2	Less translucent dentin or cavity detectable	2	25–99	2	91–100
	5	Distinct cavitation with dentin exposition	4	Radiolucency restricted to the inner half of the dentin						

### Near-infrared light transillumination

NIRT was conducted with a Diagnocam camera system (KaVo, Biberach, Germany) on dried occlusal surfaces. The light source of the dental unit was switched off, and the images from the corresponding occlusal surfaces were captured with the KID software (KaVo Integrated Desktop/version 2.4.1.6374, KaVo, Biberach, Germany) installed on a laptop. Analysis of the images was performed twice by two experienced dentists (FL and KH) according to the following criteria: no less translucent spots, caries visible limited to the enamel and no less translucent dentin, less translucent dentin or cavity visible [24]. Divergent results were discussed until a consensus finding was reached.

### Laser fluorescence measurements

LF was performed using a DIAGNOdent Pen device (KaVo, Biberach, Germany). The device was regularly calibrated according to the manufacturer’s instructions. Furthermore, the rounded glass tip of the device was individually adjusted to the autofluorescence of the tooth at a healthy dental area after brief air drying. Measurements of the occlusal surface were then made. The maximum LF reading (0–99) was recorded, and the measurements were defined as follows: 0–12 sound, 13–24 enamel and 24–99 dentin involvement [25].

### Alternating current impedance spectroscopy

ACIS was conducted with the CarieScan Pro device (orange dental, Biberach, Germany) on air-dried molar teeth and fissures isolated with cotton rolls. The ACIS readings were defined according to the following thresholds: 0–20 sound, 21–90 demineralized enamel and 91–100 dentin involvement [25].

### Treatment decision, validation and definition of the reference standard

After the clinical assessment using the different diagnostic methods as described above, a management strategy was determined for all 96 molar teeth. This management included surface-related factors, e.g., the extent of caries in relation to the pulp, presence of (micro-) cavitation and caries activity as well as the overall caries risk of each subject [5]. All steps were pre-discussed in the study group and finally agreed to by each patient. To make the independent reference standard more powerful, VE and BWR were evaluated by three different examiners (JK, FL and KH) as described above.

The hybrid reference standard consisted of two different procedures in relation to the diagnostic findings to meet the ethical requirement of an in vivo analysis. The samples undergoing non-operative validation were evaluated as healthy or occlusal surfaces with a non-cavitated carious lesion according to the findings of VE and BWR, which did not justify any restorative intervention (N = 56). The lesions without the need for operative care were integrated into an individual prophylaxis and monitoring concept. The other group, undergoing operative intervention, consisted of samples that exhibited the indication for restorative care, which was conducted at a separate appointment a maximum of two weeks after diagnosis (N = 40). For operative validation, carious dentin was removed using restrictive and selective caries removal techniques [26]. Soft dentin beneath the pulp was excavated with a self-limiting polymer bur (P1, Komet, Lemgo, Germany). The assignment at the reference values was conducted immediately after caries excavation according to the listed scores in Table 1 by one examiner (FL). After excavation and clinical judgement, the cavity was photographed for later independent reassessment. Finally, the cavity was restored with an adhesively bonded restoration (Syntac classic, Vivadent, Schaan, Lichtenstein; SONICfill, KaVo, Biberach, Germany; SonicFILL, West Collins, Orange, CA, USA). All patients were consistently informed about appropriate home-based preventive measures and were offered risk-related professional preventive dental care aiming at lowering caries activity and risk. All treatment decisions were made in cooperation with all examiners (JK, FL and KH) throughout the study period.

### Training and calibration of all diagnostic methods and the validation process

The examiners (FL and KH) underwent two-day theoretical and practical training for all diagnostic procedures used in this study (VE, BWR, NIRT, LF and ACIS) under the guidance of an experienced dentist (JK). The training included information pertaining to the study design, indices, diagnostic principles of all methods and validation procedure. The examiners then evaluated a new set of bitewing radiographs and NIRT images in the trainer's presence while discordant findings were immediately discussed, and a consensus diagnosis was reached. Subsequently, the reliability between and within the examiners (FL and KH) was determined based on 50 case examples of BWR and NIRT, and an inter-/and intra-examiner agreement of more than 90% was achieved (linear weighted kappa analysis). The training was completed by a clinical training course, during which the examiner (FL) performed clinical examinations using all diagnostic methods (VE, BWR, NIRT, LF, and ACIS) and validated ten carious dentin lesions according to the study protocol under supervision (JK).

### Statistical analysis

After data entry using a spreadsheet (Microsoft Excel, Version 16.36), statistical analysis was conducted using the statistical software SPSS (IBM SPSS Statistics for Windows, Version 25.0, Armonk, NY, USA) and R [27]. Diagnostic results from the test methods or their combinations with VE were cross-classified with the findings from the hybrid reference standard using the predefined definitions in Table 1. Overall accuracy was calculated as the percentage of correctly classified decisions (TP + TN)/(TP + TN + FP + FN), where TP, FN, FP and TN represent the counts of true positives, false negatives, false positives and true negatives, respectively. In addition to descriptive data analysis, contingency tables for cross-classification and calculation of SE and SP were done [28]. These procedures were consistently performed for all test methods and their combination using three diagnostic thresholds. These threshold values were, as shown in Table 1, enamel caries and dentin caries but also overall caries, which includes dentin caries and enamel caries together. Furthermore, the area under the receiver operating characteristic (AUROC) was calculated, and multiple comparisons between the AUROC from different methods and thresholds were conducted [29]. To interpret the AUROC, the classification by Hosmer and Lemeshow [30] was applied: AUROC value 0.5–0.7 = poor to fair discrimination; AUROC value of 0.7–0.8 = acceptable discrimination; AUROC value of 0.8–0.9 = excellent discrimination and AUC ≥ 0.9 = outstanding discrimination. If the area under the AUROC was 0.50, the model did not discriminate. The inter-/and intra-examiner reliability values were calculated using linear weighted Cohen’s kappa, where a 1-category difference could be considered as less severe than a 2-category difference. Weights ranged from 0 to 1, and the weight for cells where the raters disagreed exactly equalled 1. For cells in the lower left or upper right corners with the largest disagreement, the weight equalled 0. Each weight (W) for any cell was calculated by the formula Wxy = 1 − (|x − y|)/z, with x and y being the categories and z the total number of categories. Kappa values were categorized as poor (< 0.00), slight (0.00–0.20), fair (0.21–0.40), moderate (0.41–0.60), substantial (0.61–0.80), and almost perfect agreement (0.81–1.00) [31–33].

## Results

**Fig. 1:**
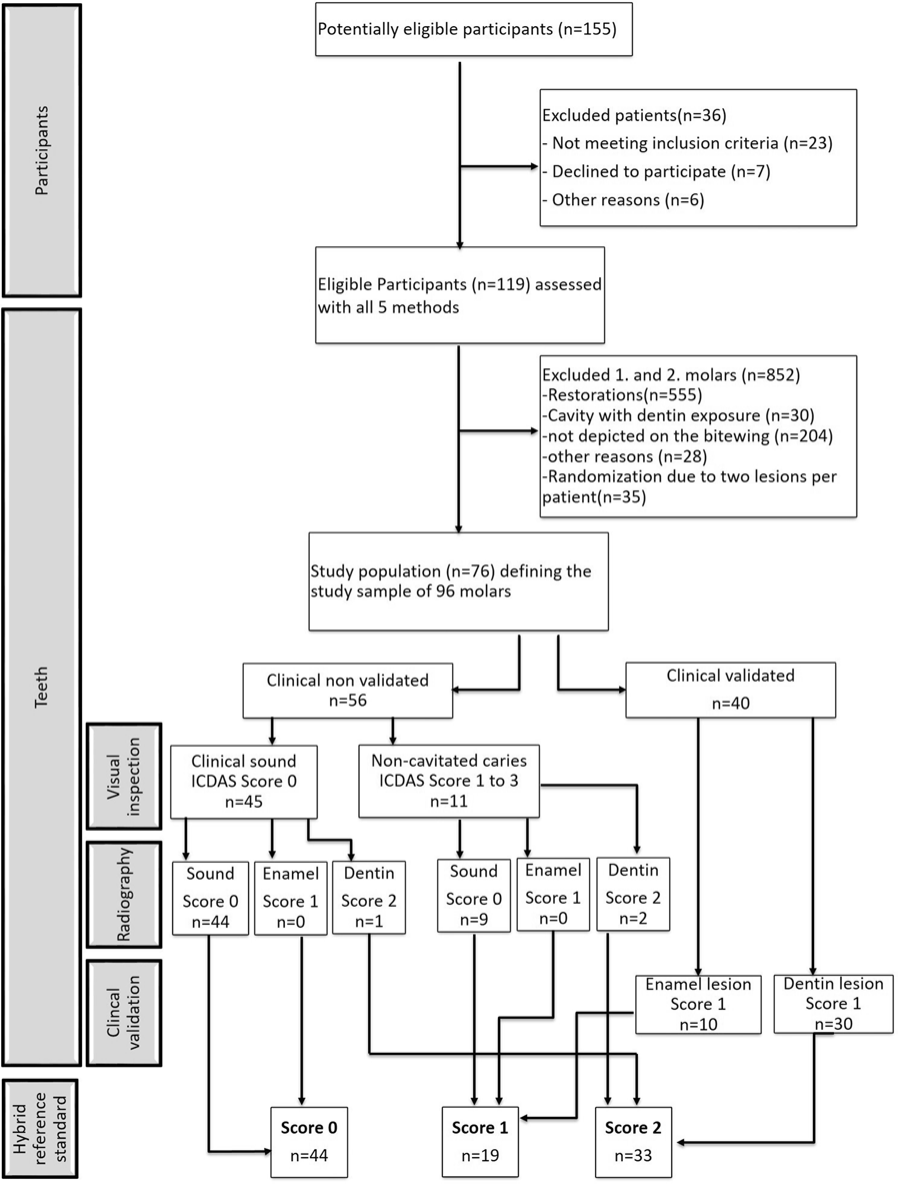
Flow diagram through the process of this diagnostic study

Out of 155 patients evaluated for eligibility, 76 participants with 96 occlusal surfaces met the inclusion criteria (Fig. 1). A maximum of two teeth per patient were randomly selected for statistical analysis. The median age of the study participants was 24.2 years (range 14–49, 6 adolescents and 70 adults, 45 women and 31 men), and their caries prevalence was moderate according to WHO criteria (5.9 DMFT and 11.2 DMFS). A total of 45.8% (N = 44) of the relevant occlusal surfaces were found to be caries-free, 19.8% (N = 19) were restricted to the enamel and 34.4% (N = 33) reached the dentin according to the hybrid reference standard (Table 2). Cross-classifications for findings of each diagnostic test method and its combination with VE in relation to the hybrid reference standard can be taken from Table 2. Overall accuracy for the two reference methods were 65.6% for VE and 69.8% for BWR. For the test methods, overall accuracy were 50.0% for NIRT, 53.1% for LF and 74.0% for ACIS. Outcome measures for diagnostic performance are summarized in Table 3 and the highest values in terms of SE and SP are highlighted in relation to the threshold used. The main results in terms of the diagnostic performance are as follows: LF and ACIS exhibited high SE for caries in general (0.77/0.75) and moderate for dentin caries (0.76/0.64), while SE for enamel caries was low (0.11/0.37). Both methods demonstrate high values of SP at all three diagnostic thresholds. NIRT showed lower values of SP at the overall caries and the dentin caries threshold (0.27/0.67) but associated with excellent SE values (1.00/0.97). For enamel caries NIRT exhibited low SE (0.21) accompanied by moderate SP (0.65). The best diagnostic performance of enamel caries detection was achieved by ACIS (SE 0.84/SP 0.84) followed by LF (SE 0.53/SP 0.92), both as adjunct methods to VE.

**Table 2:** Cross-table for comparison of all findings of visual examination (VE), bitewing radiography (BWR), laser fluorescence measurement (LF), near-infrared transillumination (NIRT) and alternating current impedance spectroscopy (ACIS) at three diagnostic thresholds

		Reference standard			
		No caries	Enamel caries	Dentin caries	Total
*Diagnostic test method*					
VE	No caries	44	0	3	47
	Enamel caries	0	19	30	49
	Dentin caries	0	0	0	0
BWR	No caries	44	19	10	73
	Enamel caries	0	0	0	0
	Dentin caries	0	0	23	23
NIRT	No caries	12	0	0	12
	Enamel caries	26	4	1	31
	Dentin caries	6	15	32	53
LF	No caries	44	8	4	56
	Enamel caries	0	2	4	6
	Dentin caries	0	9	25	34
ACIS	No caries	43	9	4	56
	Enamel caries	1	7	8	16
	Dentin caries	0	3	21	24
VE/BWR	No caries	44	0	0	44
	Enamel caries	0	19	10	29
	Dentin caries	0	0	23	23
VE/NIRT	No caries	12	0	0	12
	Enamel caries	26	4	1	31
	Dentin caries	6	15	32	53
VE/LF	No caries	44	0	2	46
	Enamel caries	0	10	6	16
	Dentin caries	0	9	25	34
VE/ACIS	No caries	43	0	1	44
	Enamel caries	1	16	11	28
	Dentin caries	0	3	21	24
Total		44	19	33	96

**Table 3:** Values for sensitivity (SE), specificity (SP) and the area under the receiver operating characteristics (AUROC) for visual examination (VE), bitewing radiography (BWR), laser fluorescence measurement (LF), near-infrared transillumination (NIRT) and alternating current impedance spectroscopy (ACIS) with and without visual examination (VE) are calculated at three diagnostic thresholds

Method	SE	SP	AUROC
*Caries detection*			
VE	**0.94 (0.88–1.01)**	**1.00 (1.00–1.00)**	**0.97 (0.93–1.00)**
BWR	0.44 (0.31–0.58)	**1.00 (1.00–1.00)**	0.72 (0.62–0.82)
NIRT	**1.00 (1.00–1.00)**	0.27 (0.14–0.40)	0.64 (0.52–0.75)
LF	0.77 (0.65–0.88)	**1.00 (1.00–1.00)**	*0.89 (0.81–0.96)*
ACIS	0.75 (0.63–0.87)	**0.98 (0.93–1.02)**	*0.86 (0.79–0.94)*
VE/BWR	**1.00 (1.00–1.00)**	**1.00 (1.00–1.00)**	**1.00 (1.00–1.00)**
VE/NIRT	**1.00 (1.00–1.00)**	0.27 (0.14–0.40)	0.64 (0.52–0.75)
VE/LF	**0.96 (0.91–1.01)**	**1.00 (1.00–1.00)**	**0.98 (0.95–1.00)**
VE/ACIS	**0.98 (0.94–1.02)**	**0.98 (0.93–1.02)**	**0.98 (0.95–1.00)**
*Enamel caries detection*			
VE	**1.00 (1.00–1.00)**	0.61 (0.50–0.72)	*0.81 (0.72–0.89)*
BWR	0.00 (0.00–0.00)	**1.00 (1.00–1.00)**	0.50 (0.35–0.65)
NIRT	0.21 (0.03–0.39)	0.65 (0.54–0.76)	0.43 (0.29–0.57)
LF	0.11 (− 0.03 to 0.24)	**0.95 (0.90–1.00)**	0.53 (0.38–0.68)
ACIS	0.37 (0.15–0.59)	*0.88 (0.81–0.95)*	0.63 (0.47–0.78)
VE/BWR	**1.00 (1.00–1.00)**	*0.87 (0.80–0.95)*	**0.94 (0.89–0.98)**
VE/NIRT	0.21 (0.03–0.39)	0.65 (0.54–0.76)	0.43 (0.29–0.57)
VE/LF	0.53 (0.30–0.75)	**0.92 (0.86–0.98)**	0.72 (0.58–0.87)
VE/ACIS	*0.84 (0.68–1.01)*	*0.84 (0.76–0.93)*	*0.84 (0.74–0.95)*
*Dentin caries detection*			
VE	0.00 (0.00–0.00)	**1.00 (1.00–1.00)**	0.50 (0.38–0.62)
BWR	0.70 (0.54–0.85)	1.00 (1.00–1.00)	*0.85 (0.75–0.95)*
NIRT	**0.97 (0.91–1.03)**	0.67 (0.55–0.78)	*0.82 (0.73–0.90)*
LF	0.76 (0.61–0.90)	0.86 (0.77–0.94)	*0.81 (0.71–0.91)*
ACIS	0.64 (0.47–0.80)	**0.95 (0.90–1.00)**	0.79 (0.69–0.90)
VE/BWR	0.70 (0.54–0.85)	**1.00 (1.00–1.00)**	*0.85 (0.75–0.95)*
VE/NIRT	**0.97 (0.91–1.03)**	0.67 (0.55–0.78)	*0.82 (0.73–0.90)*
VE/LF	0.76 (0.61–0.90)	*0.86 (0.77–0.94)*	*0.81 (0.71–0.91)*
VE/ACIS	0.64 (0.47–0.80)	**0.95 (0.90–1.00)**	0.79 (0.69–0.90)

Values greater than 0.90 are marked in bold, and values greater than 0.80 are marked in italics

All methods—NIRT, LF and ACIS—consistently showed an AUROC above 0.79 for dentin caries detection (Table 3). Comparing the AUROCs within the dentin threshold, all methods discriminated equally acceptable to excellent (0.79–0.82). In contrast, NIRT did not discriminate for the enamel threshold (0.43) and LF and ACIS discriminated poor to fair (0.53/0.63). An improvement towards acceptable discrimination could be found for the combination of LF and ACIS with VE (0.72/0.84), but not for the combination of NIRT with VE (0.43) (Table 3). Comparing the ROC curves within the enamel threshold, NIRT discriminated significantly poorer than all other methods (*p* < 0.005), especially if they were combined with VE (Table 4). For the overall caries threshold, NIRT and NIRT combined with VE also showed the weakest discrimination (0.64). Inter-examiner reliability was almost perfect for BWR with 0.89 (CI 0.79–1.00) at the first and 0.88 (CI 0.77–0.98) at the second evaluation cycle as well as for NIRT with 0.84 (CI 0.75–0.93) at first and 0.94 (CI 0.89–1.00) at the second evaluation cycle. Intra-examiner reliability was substantial to almost perfect by both examiners and methods with 0.93 (CI 0.85–1.01) and 0.92 (CI 0.84–1.01) for BWR and 0.75 (CI 0.64–0.85) and 0.84 (CI 0.75–0.93) for NIRT.

**Table 4:** Multiple comparisons between the AUC values of visual examination (VE), bitewing radiography (BWR), near-infrared transillumination (NIRT), laser fluorescence measurement (LF) and alternating current impedance spectroscopy (ACIS) with and without visual examination (VE) are calculated at three diagnostic thresholds

AUROC difference	VE	BWR	NIRT	LF	ACIS	VE/BWR	VE/NIRT	VE/LF	VE/ACIS
*Caries detection threshold*									
VE	–	**0.25**	**0.34**	0.09	0.11	0.03	**0.34**	0.00	0.01
BWR		–	0.09	**0.16**	0.13	**0.28**	0.09	**0.26**	**0.26**
NIRT			–	**0.25**	**0.23**	**0.36**	0.00	**0.34**	**0.34**
LF				–	0.02	**0.12**	**0.25**	0.10	0.09
ACIS					–	**0.14**	**0.23**	**0.12**	**0.12**
VE/BWR						–	**0.36**	0.02	0.02
VE/NIRT							–	**0.34**	**0.34**
VE/LF								–	0.00
VE/ACIS									–
*Enamel caries detection threshold*									
VE	–	**0.31**	**0.38**	**0.28**	**0.18**	**0.13**	**0.38**	0.08	0.04
BWR		–	0.07	0.03	0.13	**0.44**	0.07	**0.22**	**0.34**
NIRT			–	0.10	**0.20**	**0.51**	0.00	**0.29**	**0.41**
LF				–	0.10	**0.41**	0.10	**0.20**	**0.32**
ACIS					–	**0.31**	**0.20**	0.10	**0.22**
VE/BWR						–	**0.51**	**0.21**	0.09
VE/NIRT							–	**0.29**	**0.41**
VE/LF								–	0.12
VE/ACIS									–
*Dentin caries detection threshold*									
VE	–	**0.35**	**0.32**	**0.31**	**0.29**	**0.35**	**0.32**	**0.31**	**0.29**
BWR		–	0.03	0.04	0.05	0.03	0.03	0.04	0.05
NIRT			–	0.01	0.02	0.00	0.00	0.01	0.02
LF				–	0.01	0.04	0.01	0.00	0.01
ACIS					–	0.05	0.02	0.01	0.00
VE/BWR						–	0.03	0.04	0.05
VE/NIRT							–	0.01	0.02
VE/LF								–	0.01
VE/ACIS									–

Statistically significant values are marked in bold

## Discussion

The main objective of this in vivo diagnostic study with validation was to compare different diagnostic methods for occlusal caries detection and diagnostics at different diagnostic thresholds. It was initially hypothesized that all methods would reveal similar diagnostic performance. According to the results (Tables 2, 3, 4), the initially formulated null hypothesis must be rejected because the test methods showed heterogeneous diagnostic performance. To our knowledge, no other clinical trial has combined the comparison of the diagnostic performance of these three test methods, NIRT, LF and ACIS, at different diagnostic thresholds in one clinical analysis. The strength of this study is that permanent first and second molars were analysed in a predominately homogenous group of young adult or adolescent patients and that all test methods were applied under standardized clinical conditions.

The participants were evaluated and screened for study eligibility by the authors (JK and FL) before study entry. The study population included adolescents and young adults with complete permanent dentition and at least one molar without restoration, but only a sub-selection of these patients and their molars were finally included for statistical analysis. It must be reasoned that the included participants and their teeth may not be a representative sample of the targeted population and the generalisability of the study results must be regarded in this context. It can also be argued that the assumption of a caries prevalence, which is the basis of this study, is not representative for the targeted population. The following arguments justify our assumption of a 50% caries prevalence. Epidemiological data for the caries prevalence in Germany are merely available but based on oral health studies caries prevalence in 12-year-olds is 25.2% and in 35–45-year-olds is 97.5% [34]. Epidemiological surveys are usually based on clinical examinations of the teeth, which evaluate lesions from an advanced, clearly visible stadium while non-cavitated and/or initial lesions, as relevant in our study, mostly remain underestimated [11]. It is therefore a representative scenario for the targeted population of young adults to assume a caries prevalence of unrestored or sealed occlusal surfaces of molars with an ICDAS score of > 1 of 50% [35–37].

Further, the study data have a clustered nature because in 26% of all cases two samples per participant were included in the evaluation. Since the structure of the enamel and dentin tissue of each subject is individual, this can influence the optical properties of the teeth and thus, this may have an impact on the results. If we had opted for only one tooth per subject, the samples size would have been reduced to 76, which in turn would have lowered the statistical significance of this analysis. Future studies should include a complete cluster analysis.

Considering the cross-tabulation at the dentin caries threshold, the high rate of false-negative findings for VE becomes apparent (Table 2). Previous studies confirmed these findings with weak values for SE and strong values for SP for the detection of non-cavitated dentin lesions [38, 39]. In this study, dentin lesions were not identified visually as such, because the lesions were non cavitated and predominately hidden. This explains the relatively weak values for accuracy of 65% for visual inspection in this study. This distribution of diagnostic potential is complemented by BWR, which has its strength especially in the detection of hidden dentin lesions. By excluding cavitated lesions (ICDAS Score > 3) from the study population, we were able to better demonstrate the strength of the auxiliary test methods to detect non-cavitated dentin caries. On the other hand, the analysis incorporates an incomplete caries spectrum in the sample. This limits the generalisability of the present study, as additional thresholds, e.g., other dentin caries levels, are not proved [40–42].

The type of reference standard used in this investigation provides a solution strategy for a common and well-known problem in clinical diagnostic studies. The use of an independent and rigorous reference standard [43], e.g., histology, microradiography or µCT, is not feasible in clinical investigations as it excludes sound surfaces, non-cavitated lesions or those caries stages that can be managed by non-operative measures—shortly all lesions without the indication for operative care. With the aim of overcoming this methodological disadvantage, a hybrid reference standard was used in the present study. Although this model of a reference test meets the ethical and clinical requirements, it bears the risk of sample bias. It includes information from the test methods, which contradicts the principle of independence of index and reference tests, and therefore the present study is not free of any incorporation bias. The information about the diagnostic performance of VE as an index test is limited. The focus must be on the true index tests without intersection with the reference standard, NIRT, LF and ACIS. Nevertheless, other groups have also constructed a reference standard by including results from the index tests [44–46]. The reference standard for those samples that did not undergo operative validation is formed by VE and BWR, while the reference standard for all samples that required operative intervention is drawn from the results of the validation process. This model of a hybrid reference standard increases the risk of differential verification bias, as not all samples are subjected to the same reference standard.

Most of the results of this clinical study are in line with previous diagnostic studies concerning the methods VE, BWR and LF [16, 47–49]. We chose magnification 2.5X to achieve optimal results of visual inspection in this study. It must be considered that a visual assessment performed with unaided eyes would probably have resulted in lower values of SE and SP for VE [50]. This study additionally provides new diagnostic findings on NIRT and ACIS, which have not previously been reported in the literature [14, 15, 18–21].

Alternating current impedance spectroscopy showed strong overall accuracy values of 74% but did not show particularly well diagnostic performance for either the enamel or dentin caries detection. This renders the evaluation and assessment of the method from a clinical point of view more difficult. At the overall caries and the enamel threshold LF and ACIS show a similar range of competence, while NIRT shows significantly weaker performance than the other methods and their combinations with VE (Tables 3, 4), as well as the lowest accuracy values of only 50.0%. Additionally, NIRT was more sensitive than specific at the overall caries detection threshold, in contrast to the other methods (Table 3). The use of NIRT led to an increased number of false-positive diagnoses (Table 2). These misinterpretations may have been caused by occlusal staining of healthy molars and are described by a previous in vitro study [20]. Regarding the detection of enamel caries, all diagnostic methods revealed insufficient diagnostic performance, which was mainly caused by low values of SE (Table 3). However, as auxiliaries to VE, ACIS and especially LF increase their diagnostic potential to detect enamel lesions. Both methods seem to complement the high sensitivity for enamel lesions of ICDAS. One main result of this study is the high diagnostic performance of all three auxiliary methods for the detection of dentin caries. This fact is very important for everyday clinical practice, as the use of these diagnostic methods can support the clinician to detect lesions in dentin. However, due to the numerous limitations listed here, the test methods cannot be recommended for occlusal caries detection in general. Finally, it is important to emphasize that the present study investigated the performance of different diagnostic methods and their combination with VE in relation to anatomical-based hard tissue structures, and it did not investigate recently suggested thresholds for operative intervention. Relevant thresholds from the clinician point of view are first caries in the middle third of dentin [40], second caries in the outer fifth of dentin [41] or third caries reaching the inner quarter of dentin [42]. Here, as shown in Table 3, the hypothesis can be made that different thresholds are associated with different diagnostic performance data. It must therefore be clearly stated that the shown data should not be transferred unconditionally to other clinical situations and that further research is needed to test the diagnostic accuracy in relation to thresholds for operative interventions.

## Conclusions

All three test methods, NIRT, LF and ACIS, revealed its individual strength and limitations, but none of them exhibited impeccable diagnostic performance and is generally recommendable for occlusal caries detection. Laser fluorescence measurement and ACIS show an increase in diagnostic performance as adjunct methods to visual examination. All methods are helpful diagnostic tests to detect non-cavitated caries in dentin.

## Data Availability

The data sets generated and/or analysed during the current study are not publicly available. They consist of extensive tables, analyses and forms that contain information beyond the facts published in this analysis. Targeted data can be obtained upon request from the first author (FL).

## Abbreviations

NIRT: Near-infrared transillumination
LF: Laser fluorescence measurement
ACIS: Alternating current impedance spectroscopy
VE: Visual examination
BWR: Bitewing radiography
SE: Sensitivity
SP: Specificity
ICDAS: International Caries Detection and Assessment System II
AUROC: Receiver operating characteristic curve
AUC: Area under the curve
ASA: American Society of Anaesthesiologists
TP: True positives
FN: False negatives
FP: False positives
TN: True negatives
WHO: World Health Organization
DMFT: Decayed/missing/filled teeth
DMFS: Decayed/missing/filled surfaces
CI: Confidence interval
